# Living alone vs. living with someone as a predictor of mortality after a bone fracture in older age

**DOI:** 10.1007/s40520-020-01511-5

**Published:** 2020-03-09

**Authors:** Kaisa Koivunen, Elina Sillanpää, Mikaela von Bonsdorff, Ritva Sakari, Katja Pynnönen, Taina Rantanen

**Affiliations:** 1 Faculty of Sport and Health Sciences and Gerontology Research Center, University of Jyväskylä, Jyväskylä, Finland; 2 Folkhälsan Research Center, Helsinki, Finland

**Keywords:** Social networks, Social support, Resilience, Health stressors, Living arrangement

## Abstract

**Background:**

Living alone is a risk factor for health decline in old age, especially when facing adverse events increasing vulnerability.

**Aim:**

We examined whether living alone is associated with higher post-fracture mortality risk.

**Methods:**

Participants were 190 men and 409 women aged 75 or 80 years at baseline. Subsequent fracture incidence and mortality were followed up for 15 years. Extended Cox regression analysis was used to compare the associations between living arrangements and mortality risk during the first post-fracture year and during the non-fracture time. All participants contributed to the non-fracture state until a fracture occurred or until death/end of follow-up if they did not sustain a fracture. Participants who sustained a fracture during the follow-up returned to the non-fracture state 1 year after the fracture unless they died or were censored due to end of follow-up.

**Results:**

Altogether, 22% of men and 40% of women sustained a fracture. During the first post-fracture year, mortality risk was over threefold compared to non-fracture time but did not differ by living arrangement. In women, living alone was associated with lower mortality risk during non-fracture time, but the association attenuated after adjustment for self-rated health. In men, living alone was associated with increased mortality risk during non-fracture time, although not significantly.

**Conclusion:**

The results suggest that living alone is not associated with pronounced mortality risk after a fracture compared to living with someone.

**Electronic supplementary material:**

The online version of this article (10.1007/s40520-020-01511-5) contains supplementary material, which is available to authorized users.

## Introduction

Humans are by nature social creatures and it is widely recognized that social networks are associated with health outcomes. According to the conceptual model of Berkman et al. [1], social networks operate through different psychosocial mechanisms, such as social support and social engagement, which influence health. Further, these mechanisms influence more proximate pathways to health status, such as health behavioral, psychological and physiological pathways. Living arrangements may have a substantial impact on the psychosocial mechanisms affecting health, such as the availability of social support. The number of older people living alone is rising in most countries, primarily owing to population aging, widowhood, modernization and cultural transitions, individual values, and the availability of social services [2]. In line with the conceptual model of Berkman et al. [1], studies have indicated that living alone may predispose to social vulnerability such as social isolation and loneliness [3, 4], which in turn correlate with increased likelihood of adverse health behavior, higher blood pressure, and markers of inflammation [5, 6] as well as higher mortality risk [7, 8].

Older people living in single households may be particularly vulnerable when their need for support in managing daily tasks increases with aging [2]. Hence, the consequences of sudden catastrophic health events may be more severe among older people living alone and lead to an increased need for health and social care services. Living with someone may provide emotional and practical social support [9], which have been recognized as important resources for resilience in older age [10]. Resilience refers to the capacity to adapt or recover mentally and physically in the face of adversity [11, 12], and is likely to be one of the key factors supporting positive aging trajectories and survival when faced with stressful events in older age [13, 14].

Bone fractures are common catastrophic adverse health events in old age and induce acute physiological and psychosocial stress. Fractures increase the risk for health decline and premature mortality [15–17]. Earlier studies have reported contradictory findings on how psychosocial factors, including social support and living arrangements, affect health outcomes following a fracture. Adequacy of post-fracture social support or a higher number of pre-fracture social contacts have been associated with better recovery and lower mortality after a fracture [18, 19]. Other studies have found no association between social support and recovery or between living arrangement and survival after hip fracture [20, 21]. These inconsistent findings may be explained by differences in the measures of social support used and timing of the observations (before vs. after fracture). However, studies with other patient groups, such as patients suffering from acute myocardial infarction [22, 23] or ischemic stroke [24] have found that living alone is associated with increased mortality risk after acute health events.

This study investigated whether living arrangement in old age is associated with mortality risk after a bone fracture and whether the potential association is different compared to mortality risk during time without fracture. Increased mortality risk after fracture among those living alone would suggest that living alone increases vulnerability and decreases the likelihood of recovery when confronted with an acute adverse health event.

## Methods

### Study design

The study sample comprised participants from the Evergreen Study, which has been described in detail elsewhere [25]. In brief, the study was conducted between 1989 and 1990 in Jyväskylä, Finland. All the residents aged 75 in 1989 and those aged 80 in 1990 formed the target group. In total, 617 persons took part in the study. Of this study population, 190 men and 409 women were community living and formed the sample for this study. Fracture incidence and mortality were followed up for 15 years after baseline.

### Living arrangement

At baseline, living arrangement was defined as living alone versus living with someone (a partner or another adult, e.g., family member). For the sensitivity analyses, we collected information on possible changes in living arrangements 5 years after the baseline. This information was available for 423 participants (95% of the survivors).

### Ascertainment of fractures and death

Fracture incidence and mortality were followed from the beginning of 1990 until the end of April 2005. Information on the ICD-10 diagnosis code, date, scene and follow-up treatment of the fracture were obtained from patient records kept by the local health centers in the health care district and in the Central Hospital of Central Finland, where all the participants’ fractures were treated. Death dates were obtained from the Population Register of Finland. Fractures of toes and fingers were excluded from the analyses. Fractures were categorized by location into proximal fractures (hip, pelvis and lumbar spine) and distal fractures (thoracic and cervical spine, upper extremity, lower leg and foot, head and collar bone). For participants who sustained at least one proximal fracture, the date of the first proximal fracture was chosen while for participants who sustained distal fractures only, the date of the first distal fracture was chosen. Follow-up treatment after fracture was categorized as no follow-up treatment or treatment in an outpatient clinic versus treatment in a hospital ward.

### Covariates

At baseline, information regarding participants’ sociodemographic characteristics, health determinants and psychosocial well-being was obtained in interviews using standardized questionnaires. Sociodemographic items included age, sex, marital status and years of education. Marital status was categorized as married, single, divorced or widowed. Educational background was recorded as years of full-time education. Self-rated health was assessed with the single question: “How would you yourself describe your health during the last year?” with five response options. For statistical analysis, we categorized responses as good, moderate and poor. Level of everyday physical activity was studied by a single six‐category question where the respondent chooses the option that best describes his/her typical level of physical activity [26]. Participants whose self-reported amount of weekly physical activity did not meet the needed level of national physical activity guidelines for older adults (at least 2.5 h of moderate activity or at least 1.25 h of vigorous activity per week) [27] were assigned to the lower physical activity group. Smoking status was classified according to whether the participant had ever been a smoker or not. Number of chronic conditions was calculated from self-reports and ascertained in a subsequent clinical examination by a physician.

Factors indicating psychosocial well-being included loneliness, warmth of the spousal relationship, number of close friends and depressive symptoms. Loneliness was measured using a single structured item with four response options. Those who reported often or almost always feeling lonely were categorized as lonely. Warmth of the spousal relationship was assessed with the question: “How close do you feel your relationship with your partner is?” were categorized the response options as “not in a relationship”, “not very close” and “close”. Participants were also asked to report their number of close friends. For statistical analysis, we categorized the responses into three categories as follows: “no friends”, “1–3 friends” and “more than 3 friends”. Depressive symptoms were assessed using the 20-item Center for Epidemiologic Studies Depression Scale (CES-D) with the cutoff value of ≥ 16 for increased risk of depressive symptoms [28].

### Statistical analysis

We compared baseline characteristics between participants who were living alone and those living with someone at baseline using cross-tabulations and Chi-square tests of significance for categorical variables and *t*-tests for continuous variables. Univariate Cox regression models were carried out to examine associations between baseline characteristics and mortality risk across the entire follow-up time.

**Fig. 1:**
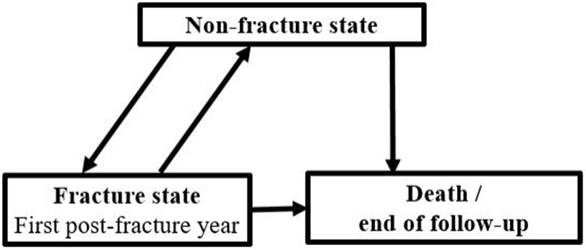
An extension of the illness–death model used in the analysis

Mortality risk with and without fracture was analyzed with Cox regression analysis using an extension of the illness–death model [29]. A time-fixed exposure variable does not usually meet the proportional hazard assumption, as the risk for death is highest immediately after the injury and attenuates during the following years [30]. In our model, fracture states were modeled as a time-dependent variable in a relative risk model based on a counting process formulation (Fig. 1). All participants contributed to the non-fracture state until a fracture occurred or until death or end of follow-up if they did not sustain a fracture. Participants, who sustained a fracture, were assigned to the fracture state for the first post-fracture year. These subjects re-assigned to the non-fracture state after the first post-fracture year unless they died or they were censored due to the end of follow-up during the 1-year period. The main effects of living alone indicate the mortality risk compared to living with someone and the main effects of fracture state indicate the mortality risk compared to non-fracture state. Interaction terms between living alone and fracture state were used to investigate whether the association between living alone and mortality risk is different in fracture state (during the first post-fracture year) compared to the association in non-fracture state (other time periods in the follow-up).

The associations of living arrangements and fractures with mortality risk adjusted for age at baseline were analyzed first in the basic model. Model 2 was adjusted for age and loneliness, and model 3 for age, loneliness and self-rated health. Covariates were selected based on their potential as confounders. The selected covariates were all associated with both the predictor (living arrangement) and outcome (mortality) in our data.

The analyses were conducted separately for men and women, as the association between living arrangement and mortality risk varies by sex [22, 31–33]. *P*-values less than 0.05 were considered statistically significant. Analyses were performed using SPSS Statistics 24 for Windows and R version 3.5.1.

## Results

At baseline, 44 men (23%) and 247 (67%) women lived alone. Among both sexes, participants who lived alone were older and more likely to be widowed (Table 1). Men living alone more often reported their health as poor and good, whereas men living with someone more often reported their health as moderate. Participants living alone did not report more loneliness than those living with another person. One (1%) of the men and 52 (39%) of the women living with someone lived with someone other than a partner at baseline.

**Table 1:** Characteristics of the participants by sex and living arrangement (*n* = 599)

	Men		*P*-value	Women		*P*-value
	Living alone (*n* = 44)	Living with someone (*n* = 146)		Living alone (*n* = 274)	Living with someone (*n* = 135)	
	Number (%)		*χ*^2^	Number (%)		*χ*^2^
Age, 80 vs. 75	22 (50)	48 (33)	**0.039**	131 (48)	50 (37)	**0.039**
Marital status						
Married	6 (14)	142 (97)	**< 0.001**	2 (1)	80 (59)	**< 0.001**
Single	5 (11)	2 (1)		54 (20)	9 (7)	
Divorced	8 (18)	0 (0)		28 (10)	9 (7)	
Widowed	25 (57)	2 (1)		190 (69)	37 (27)	
Spousal relationship						
Not in a relationship	35 (83)	1 (1)	**< 0.001**	264 (99)	50 (39)	**< 0.001**
Not very close	5 (12)	21 (15)		0 (0.0)	22 (17)	
Close	2 (5)	120 (85)		2 (1)	58 (44)	
Loneliness, yes	17 (40)	47 (32)	0.387	39 (29)	105 (39)	0.054
CES-D score, > 16	15 (39)	39 (29)	0.267	83 (34)	46 (36)	0.595
Number of close friends						
0	9 (21)	37 (27)	0.494	50 (19)	30 (23)	0.264
1–3	19 (45)	48 (35)		159 (60)	67 (51)	
> 3	14 (33)	51 (38)		57 (21)	34 (26)	
Self-rated health						
Good	9 (21)	19 (14)	**0.027**	44 (17)	15 (13)	0.069
Moderate	22 (52)	101 (74)		183 (69)	75 (64)	
Poor	11 (26)	17 (12)		38 (14)	28 (24)	
Physical activity, low	30 (73)	87 (64)	0.275	183 (70)	78 (67)	0.469
Smoker ever, yes	30 (71)	102 (74)	0.750	31 (12)	15 (13)	0.816
Fractures during follow-up, yes	10 (23)	32 (22)	0.910	118 (43)	46 (34)	0.081

	Mean (SD)		*t*-test	Mean (SD)		*t*-test
Number of chronic conditions	1.6 (1)	1.6 (1)	0.880	1.7 (2)	1.7 (2)	0.874
Years of education	5.6 (3)	6.4 (4)	0.116	5.9 (3)	5.8 (3)	0.735

Statistically significant values are bolded

*χ*^2^ = Chi-square test, CES-D = Center for Epidemiologic Studies Depression Scale

The follow-up encompassed 1544 person-years of surveillance among the men and 3790 person-years among the women. During the follow-up, 42 of the men and 164 of the women sustained at least one fracture and 167 men and 330 women died. Mean time from baseline to fracture was 5.3 years (SD 3.7) for men and 6.7 years (SD 4.1) for women and for 92% the main cause of the fracture was a fall. No differences in fracture events were observed between participants who lived alone and those living with someone (Table 1). In addition, no difference between fractured participants who lived alone and those living with someone were observed in either fracture site (distal vs. proximal) or follow-up treatment after fracture (no follow-up treatment or treatment in an outpatient clinic vs. a hospital ward).

The crude mortality rate for men was 10.8/100 person-years and for women 8.7/100 person-years. Of the fractured participants, 13 (31%) men and 39 (24%) women died during the first post-fracture year. The baseline characteristics of participants according to fracture status (non-fractured, survived the first post-fracture year, and died during the first post-fracture year) are presented in Supplementary Table 1. Men who died during the first post-fracture year had more often rated their health as poor than non-fractured participants and first post-fracture-year survivors. Among both sexes, participants who died during the first post-fracture-year were less physically active than the non-fractured or first post-fracture-year survivors.

Table 2 shows mortality hazards obtained with the univariate Cox regression analysis. In men, living alone compared to living with someone increased mortality risk although not significantly, while in women living alone protected against death. Among both sexes, older age at baseline, poor self-rated health, lower physical activity and higher number of chronic conditions were associated with elevated mortality risk. Furthermore, among men, being divorced and reporting loneliness increased mortality risk, whereas having a close spousal relationship compared to not having a partner was a protective factor. In women, depressiveness was associated with increased mortality risk while longer education and having more than one close friend were associated with decreased mortality risk. In addition, women who reported not having a close relationship with their partner had higher mortality risk than women without a partner (*P* = 0.050).

**Table 2:** Unadjusted hazard ratios of risk factors for mortality risk stratified by sex

	Men (*n* = 190) HR (95% CI)	Women (*n* = 409) HR (95% CI)
Age, 80 vs. 75	**1.60 (1.17**–**2.19)**	**1.83 (1.47**–**2.28)**
Living alone, yes	1.33 (0.93–1.89)	**0.75 (0.60**–**0.94)**
Marital status		
Married	1.00	1.00
Single	1.04 (0.46–2.35)	0.88 (0.61–1.25)
Divorced	**2.25 (1.10**–**4.60)**	1.16 (0.77–1.76)
Widowed	0.88 (0.61–1.25)	0.83 (0.63–1.10)
Loneliness, yes vs. no	**1.04 (1.02**–**1.94)**	1.13 (0.90–1.42)
Spousal relationship		
Not in a relationship	1.00	1.00
Not very close	0.73 (0.43–1.25)	1.56 (1.00–2.44)
Close	**0.68 (0.46**–**0.99)**	1.04 (0.76–1.42)
Number of close friends		
0	1.00	1.00
1–3	1.48 (0.98–2.24)	**0.68 (0.52**–**0.90)**
> 3	1.45 (0.96–2.21)	**0.64 (0.46**–**0.89)**
CES-D score > 16 vs. < 16	1.10 (0.78–1.56)	**1.29 (1.01**–**1.63)**
Self-rated health		
Good	1.00	1.00
Moderate	1.02 (0.66–1.59)	1.11 (0.80–1.54)
Low	**2.08 (1.20**–**3.61)**	**2.95 (1.99**–**4.35)**
Physical activity, lower vs. higher	**2.13 (1.50**–**3.03)**	**1.31 (1.02**–**1.68)**
Smoker, ever	1.40 (0.98–2.01)	1.31 (0.94–1.82)
Number of chronic conditions, per one	**1.21 (1.07**–**1.36)**	**1.13 (1.05**–**1.22)**
Education, per year	0.99 (0.94–1.04)	**0.95 (0.92**–**0.99)**

Statistically significant values are bolded

*HR* hazard ratio, *CI* confidence interval, *CES-D* Center for Epidemiologic Studies Depression Scale

Mortality risk during the first post-fracture year compared to non-fracture state was almost fourfold in men and over threefold in women after adjustment for covariates (Table 3). Table 4 shows the main effects and interactions of living arrangement and fracture state on mortality. The main effect of living alone with mortality risk indicated that in women, living alone compared to living with someone was a protective factor after adjustment for age and loneliness. However, further adjustment for self-rated health attenuated the association. In men, living alone compared to living with someone was associated with an increased mortality risk, although the estimates did not reach statistical significance. Interaction effects between living alone and fracture state were not statistically significant in either men or women. The non-significant interactions suggest that after the fracture, mortality risk was similar than during the other time periods between subjects living alone and living with someone.

**Table 3:** Mortality risk during the first post-fracture year (fracture state) compared to non-fracture state stratified by sex

	Model 1	Model 2	Model 3
	HR (95% CI)	HR (95% CI)	HR (95% CI)
	*n* = 190	*n* = 188	*n* = 179
Men			
Non-fracture state^a^	1.00	1.00	1.00
Fracture state^b^	**3.33 (1.86**–**5.94)**	**3.64 (2.03**–**6.51)**	**3.74 (2.08**–**6.70)**

	*n* = 409	*n* = 402	*n* = 379
Women			
Non-fracture state^a^	1.00	1.00	1.00
Fracture state^b^	**3.16 (2.22**–**4.37)**	**3.10 (2.21**–**4.35)**	**3.27 (2.32**–**4.63)**

Statistically significant values are bolded

Model 1: adjusted for age at baseline

Model 2: adjusted for age and loneliness at baseline

Model 3: adjusted for age, loneliness and self-rated health at baseline

*HR* hazard ratio, *CI* confidence interval

^a^Reference time; all other follow-up time, excluding the first post-fracture year of the participants sustaining a fracture

^b^The first post-fracture year of the participants sustaining a fracture

**Table 4:** Main effects and interactions of living arrangements and fracture state (the first post-fracture year) on the mortality risk stratified by sex

	Model 1	Model 2	Model 3
	HR (95% CI)	HR (95% CI)	HR (95% CI)
	*n* = 190	*n* = 188	*n* = 179
Men			
Living alone^a^	1.28 (0.89–1.85)	1.20 (0.82–1.75)	1.27 (0.86–1.87)
Fracture state^b^	**3.75 (1.94**–**7.28)**	**3.95 (2.04**–**7.67)**	**4.05 (2.08**–**7.89)**
Living alone * fracture state^c^	0.54 (0.16–1.87)	0.71 (0.18–2.77)	0.70 (0.18–2.77)

	*n* = 409	*n* = 402	*n* = 379
Women			
Living alone^a^	**0.71 (0.55**–**0.90)**	**0.70 (0.55**–**0.90)**	0.84 (0.64–1.09)
Fracture state^b^	**3.50 (1.99**–**6.14)**	**3.38 (1.92**–**5.96)**	**3.72 (2.06**–**6.73)**
Living alone * fracture state^c^	0.85 (0.42–1.69)	0.88 (0.44–1.78)	0.83 (0.40–1.72)

Statistically significant values are bolded

Model 1: adjusted for age at baseline

Model 2: adjusted for age and loneliness at baseline

Model 3: adjusted for age, loneliness and self-rated health at baseline

*HR* hazard ratio, *CI* confidence interval

^a^The main effect of the living arrangements on the mortality risk (those living alone vs. those living with someone)

^b^The main effect of fracture state on the mortality risk (the mortality risk during the first post-fracture year vs. the mortality risk during other time periods in the follow-up)

^c^The interaction of living alone and fracture state on the mortality risk

### Sensitivity analyses

We conducted sensitivity analyses by excluding participants based on factors associated with living arrangement or fracture that could influence the results (data not shown). First, we excluded participants whose living arrangement had changed 5 years after baseline. In total, 14 men and 30 women had changed from living with someone to living alone, whereas two men and 24 women had changed from living alone to living with someone. Second, we excluded participants who lived with someone other than a partner, as they may have had worse health status than those living with a partner [34]. Third, to take account of the quality of social support at home, we excluded participants who were living with a spouse and did not have very close spousal relationship. These exclusions did not materially change the results.

We considered fracture severity by excluding participants with a potentially less severe fracture. First, we excluded participants with distal fractures and then those who had either no follow-up treatment or treatment in an outpatient clinic; however, this did not change the results. Finally, we stratified participants by age group to test whether age might influence the results. Marked differences in results were not observed between the 75- and 80-year-old participants.

## Discussion

In the current community-based cohort study among 75- and 80-year-old people, mortality risk during the first year after a bone fracture increased over threefold compared to the non-fracture state, but did not differ between those who lived alone and those who lived with another person.

Inconsistent results have been reported on whether living alone increases the risk for health decline after an adverse health event [20, 22, 23]. A potential explanation for these mixed findings is that old people who live alone do not form a uniform group but consist of both socially isolated and socially integrated persons. In fact, in many modern societies older adults who live alone often have large and diverse social networks [35]. Therefore, living alone does not necessarily indicate social isolation and/or loneliness, both of which are important psychosocial mechanisms influencing health [1]. In the present study, women living on their own reported less loneliness than women living with someone, although the comparison did not quite reach statistical significance.

Our hypothesis was that among older people living with someone might be a source of social resilience compared to living alone and potentially predict better survival after a bone fracture. Fractures among older people are sometimes referred to as “the beginning of the end”, and recovery from a fracture in old age requires psychosocial and physiological resources. Although mortality does not directly describe non-recovery from fracture nor does survival indicate recovery, they are powerful indicators of health changes and can provide further understanding of health trajectories subsequent to experiencing health stressors.

In older ages, because of their longer life expectancy, women are more likely than men to be widowed and living alone. During the non-fracture time, living alone versus living with somebody was associated with lower mortality in women; however, this association was attenuated after adjustment for self-rated health, suggesting that participants who lived alone enjoyed better health. In addition, differences between men and women in their relationship preferences and the size of their support networks outside the home may partly explain the lower mortality observed among women living alone. According to Dykstra and Fokkema [36], married women tend to feel more emotional loneliness compared to married men. The authors suggested that women are more inclined to invest in relationships with friends and relatives, whereas men are more partner-centered. In our analyses, having close friends was also associated with lower mortality risk among women. Among men, in contrast, a close spousal relationship was associated with lower, and being divorced with higher, mortality risk. It is possible that, in older ages, women have greater adaptability to manage their daily life and survive living alone than men.

Some earlier studies have also found that the importance of living arrangements as a risk factor for mortality decreases with age [33, 37–39]. The authors suggest that among younger people, living alone may be experienced as a stressful psychosocial situation, whereas in older age living alone is a more normative condition, at least in Western societies [37, 39]. In the current study, the participants were 75 or 80 years old at baseline and were even older at the time of the fracture. The surviving cohort effect might also explain why, contrary to many other earlier findings with younger study populations, living alone was not associated with higher mortality risk compared to living with someone. The participants of this study present the surviving elite of their cohort, while their birth cohort members who died at a younger age could not be observed. In addition, by including also non-fractured participants, we were unable to account for the age at the fracture to test whether the increasing age influenced the association between living arrangement and mortality after fracture.

The strengths of this study include population-based epidemiological data with a long follow-up and linkages to comprehensive patient records and a register of deaths. This enabled us to include participants also in the non-fracture state and to compare the associations between participants with and without fracture exposure. Such a comparison may reveal resources for recovery that has a more pronounced role following adverse health events. Information on both psychosocial well-being and physical functioning was collected before fracture occurrence and thus was not confounded by situational factors related to the fracture. In addition, we conducted extensive sensitivity analyses to confirm the results by controlling for factors related to living arrangement and fracture event.

This study has several limitations. First, the total sample size was small, especially among men, due to fewer males in the population in the age groups targeted. The small sample size reduced the statistical power and possibilities to control for specific fracture types and to analyze gender differences. In addition, the frequency distribution shown in the descriptive analysis was affected due to small sample size among men. Second, changes in living arrangements could not be taken into account in the main analysis. However, we had access to information on the living situation of 423 participants at 5 years after baseline. The additional analysis excluding participants whose living arrangement had changed during the first 5 years of the follow-up did not alter the results. In addition, we could not consider with whom the participants were living. It has been reported that, among older men, living with a person other than one’s spouse is a risk factor for developing disability [34]. In the additional analysis, we excluded participants living with persons other than spouses, but this did not substantially change the results. The time to fracture varied among individuals, and consequently the age at fracture also varied. This may have affected the results, because older age may increase the vulnerability to mortality risk related to different living conditions. Unfortunately, age at fracture cannot be included in the model including also people who did not sustain a fracture. However, our additional subgroup analyses limited to those who sustained a fracture (not shown in the manuscript) suggested that age at fracture did not influence the difference in survival times between those living alone vs. living with someone. Finally, data on some potential confounders, such as self-perceived social support, quality of life and catastrophic health stressors other than fractures were not available.

It is important to note that the present baseline data were collected almost 30 years ago and thus may not necessarily represent social conditions in corresponding cohorts today. For instance, the proportion of older people living with someone other than a partner or living in a long-term care facility has declined dramatically [40]. Owing to a greater number of frail older people living at home with the help of home care, a fraction of the older people currently living at home may well have poorer physical and cognitive functioning than earlier cohorts. In addition, differences in life expectancy between men and women have been decreasing, a trend that is likely to affect older people’s living arrangements in the future [40]. During the next few decades, the proportion of men living alone is likely to slowly increase and the proportion of women living alone to decrease. Moreover, post-fracture treatment and rehabilitation protocols may have changed, a factor that may also affect survival rates after fractures.

In conclusion, our results suggest that living with someone may not necessarily be a resource for better survival after a fracture in 75- and 80-year-old men and women. Further studies are required to confirm this result and to study whether the impact of living arrangements differs among younger or older cohorts or after different health stressors.

## Electronic supplementary material

Below is the link to the electronic supplementary material.

Supplementary material 1 (DOCX 17 kb)
