# Supplementary material for: Living alone vs. living with someone as a predictor of mortality after a bone fracture in older age
Source: Aging Clin Exp Res. 2020 Mar 9;32(9):1697–705. doi: 10.1007/s40520-020-01511-5 (PMC7508956; doi:10.1007/s40520-020-01511-5)
Supplement: Supplementary file 1 — Supplementary material 1 (DOCX 17 kb) [file 40520_2020_1511_MOESM1_ESM.docx]

**LIVING ALONE VS. LIVING WITH SOMEONE AS A PREDICTOR OF MORTALITY AFTER A BONE FRACTURE IN OLDER AGE**

Aging Clinical and Experimental Research

Kaisa Koivunen^1^, MSc, Elina Sillanpää^1^, PhD, Mikaela von Bonsdorff^1,2^, PhD, Ritva Sakari^1^, PhD, Katja Pynnönen^1^, PhD, Taina Rantanen, PhD^1^

1. Faculty of Sport and Health Sciences and Gerontology Research Center, University of Jyväskylä, Finland

2. Folkhälsan Research Center, Helsinki, Finland

Corresponding author:

Kaisa Koivunen

e-mail: [kaisa.m.koivunen@jyu.fi](mailto:kaisa.m.koivunen@jyu.fi)

Supplementary Table 1. Baseline Characteristics of the Participants Stratified into Those Who Did Not Sustain a Fracture, Those Who Had a Fracture and Either Survived the First Post-fracture Year or Died during the First Post-fracture Year.

|  | Men | | |  | Women | | |  |
| --- | --- | --- | --- | --- | --- | --- | --- | --- |
|  | Non-fractured | Fractured, survived 1. year | Fractured, died 1. year |  | Non-fractured | Fractured, survived 1. year | Fractured, died 1. year |  |
|  | Number (%) | | | p-value | Number (%) | | | p-value |
| Living arrangement |  |  |  |  |  |  |  |  |
| Living with someone | 114 (77.0) | 22 (75.9) | 10 (76.9) | 0.991 | 89 (36.3) | 32 (25.6) | 14 (35.9) | 0.107 |
| Living alone | 34 (23.0) | 7 (24.1) | 3 (23.1) |  | 156 (63.7) | 93 (74.4) | 25 (64.1) |  |
| Age at baseline |  |  |  |  |  |  |  |  |
| 75 years | 98 (66.2) | 16 (55.2) | 6 (16.2) | 0.223 | 131 (53.5) | 79 (63.2) | 18 (46.2) | 0.091 |
| 80 years | 50 (33.8) | 13 (44.8) | 7 (53.8) |  | 114 (46.5) | 46 (36.8) | 21 (53.8) |  |
| Marital status |  |  |  |  |  |  |  |  |
| Married | 116 (78.4) | 22 (75.9) | 10 (76.9) | 0.772 | 49 (20.0) | 25 (20.0) | 8 (20.5) | 0.714 |
| Single | 5 (3.4) | 2 (6.9) | 0 (0.0) |  | 32 (13.1) | 25 (20.0) | 6 (15.4) |  |
| Divorced | 7 (4.7) | 0 (0.0) | 1 (7.7) |  | 21 (8.6) | 12 (9.6) | 4 (10.3) |  |
| Widowed | 20 (13.5) | 5 (17.2) | 2 (15.4) |  | 143 (58.4) | 63 (50.4) | 21 (53.8) |  |
| Loneliness, yes | 56 (38.1) | 5 (17.9) | 3 (23.1) | 0.081 | 78 (32.5) | 50 (40.7) | 16 (41.0) | 0.239 |
| Number of close friends |  |  |  |  |  |  |  |  |
| 0 | 35 (24.8) | 7 (28.0) | 4 (33.3) | 0.283 | 49 (20.8) | 19 (15.6) | 12 (30.8) | 0.052 |
| 1-3 | 56 (39.7) | 10 (40.0) | 1 (8.3) |  | 129 (54.7) | 81 (66.4) | 16 (41.0) |  |
| >3 | 50 (35.5) | 8 (32.0) | 7 (58.3) |  | 58 (24.6) | 22 (18.0) | 11 (28.2) |  |
| Depressive symptoms, CESD >16 | 39 (29.1) | 20 (35.7) | 5 (45.5) | 0.454 | 80 (36.2) | 36 (30.5) | 13 (36.1) | 0.561 |
| Self-rated health |  |  |  |  |  |  |  |  |
| Good | 24 (17.1) | 4 (15.4) | 0 (0.0) | **0.018** | 30 (13.3) | 23 (19.2) | 6 (15.8) | 0.065 |
| Moderate | 96 (68.6) | 20 (76.9) | 7 (53.8) |  | 148 (65.8) | 86 (71.7) | 24 (63.2) |  |
| Poor | 20 (14.3) | 2 (7.7) | 6 (46.2) |  | 47 (20.9) | 11 (9.2) | 8 (21.1) |  |
| Physical activity |  |  |  |  |  |  |  |  |
| Higher | 46 (33.1) | 13 (52.0) | 1 (7.7) | **0.021** | 65 (29.5) | 45 (38.1) | 6 (15.4) | **0.024** |
| Lower | 93 (66.9) | 12 (48.0) | 12 (92.3) |  | 155 (70.5) | 73 (61.9) | 33 (84.6) |  |
|  | Mean (sd) | | |  | Mean (sd) | | |  |
| Years of education | 6.4 (3.9) | 5.5 (3.2) | 5.7 (2.7) | 0.479 | 5.6 (3.1) | 6.1 (3.7) | 5.9 (1.9 ) | 0.350 |
| Number of chronic conditions | 1.7 (1.4) | 1.2 (1.1) | 1.8 (0.7) | 0.168 | 1.7 (1.5) | 1.7 (1.4) | 1.9 (1.49) | 0.699 |

*Notes.* Chi square test was used for categorical variables and one-way ANOVA for continuous variables. sd=standard deviation.
